# Evaluating User Experience With a Chatbot Designed as a Public Health Response to the COVID-19 Pandemic in Brazil: Mixed Methods Study

**DOI:** 10.2196/43135

**Published:** 2023-04-03

**Authors:** Bruno Azevedo Chagas, Adriana Silvina Pagano, Raquel Oliveira Prates, Elisa Cordeiro Praes, Kícila Ferreguetti, Helena Vaz, Zilma Silveira Nogueira Reis, Leonardo Bonisson Ribeiro, Antonio Luiz Pinho Ribeiro, Thais Marques Pedroso, Alline Beleigoli, Clara Rodrigues Alves Oliveira, Milena Soriano Marcolino

**Affiliations:** 1 Computer Science Department, Universidade Federal de Minas Gerais Belo Horizonte Brazil; 2 Arts Faculty, Universidade Federal de Minas Gerais Belo Horizonte Brazil; 3 Telehealth Center University Hospital Universidade Federal de Minas Gerais Belo Horizonte Brazil; 4 Telehealth Network of Minas Gerais Belo Horizonte Brazil; 5 Department of Internal Medicine, Medical School, Universidade Federal de Minas Gerais Belo Horizonte Brazil; 6 Flinders Digital Health Centre, Flinders University Adelaide Australia; 7 Caring Futures Institute, Flinders University Adelaide Australia

**Keywords:** user experience, chatbots, telehealth, COVID-19, human-computer interaction, HCI, empirical studies in human-computer interaction, empirical studies in HCI, health care information systems

## Abstract

**Background:**

The potential of chatbots for screening and monitoring COVID-19 was envisioned since the outbreak of the disease. Chatbots can help disseminate up-to-date and trustworthy information, promote healthy social behavior, and support the provision of health care services safely and at scale. In this scenario and in view of its far-reaching postpandemic impact, it is important to evaluate user experience with this kind of application.

**Objective:**

We aimed to evaluate the quality of user experience with a COVID-19 chatbot designed by a large telehealth service in Brazil, focusing on the usability of real users and the exploration of strengths and shortcomings of the chatbot, as revealed in reports by participants in simulated scenarios.

**Methods:**

We examined a chatbot developed by a multidisciplinary team and used it as a component within the workflow of a local public health care service. The chatbot had 2 core functionalities: assisting web-based screening of COVID-19 symptom severity and providing evidence-based information to the population. From October 2020 to January 2021, we conducted a mixed methods approach and performed a 2-fold evaluation of user experience with our chatbot by following 2 methods: a posttask usability Likert-scale survey presented to all users after concluding their interaction with the bot and an interview with volunteer participants who engaged in a simulated interaction with the bot guided by the interviewer.

**Results:**

Usability assessment with 63 users revealed very good scores for chatbot usefulness (4.57), likelihood of being recommended (4.48), ease of use (4.44), and user satisfaction (4.38). Interviews with 15 volunteers provided insights into the strengths and shortcomings of our bot. Comments on the positive aspects and problems reported by users were analyzed in terms of recurrent themes. We identified 6 positive aspects and 15 issues organized in 2 categories: usability of the chatbot and health support offered by it, the former referring to usability of the chatbot and how users can interact with it and the latter referring to the chatbot’s goal in supporting people during the pandemic through the screening process and education to users through informative content. We found 6 themes accounting for what people liked most about our chatbot and why they found it useful—3 themes pertaining to the usability domain and 3 themes regarding health support. Our findings also identified 15 types of problems producing a negative impact on users—10 of them related to the usability of the chatbot and 5 related to the health support it provides.

**Conclusions:**

Our results indicate that users had an overall positive experience with the chatbot and found the health support relevant. Nonetheless, qualitative evaluation of the chatbot indicated challenges and directions to be pursued in improving not only our COVID-19 chatbot but also health chatbots in general.

## Introduction

The burden on health systems during the COVID-19 pandemic reached unprecedented levels in both high- and low-income countries globally. The increase in demand for the provision of care through the several COVID-19 pandemic waves required global public health responses and challenged health care systems’ capacity as well as health units’ resilience [1]. Concomitantly, there was a sudden unprecedented demand for information and a widespread amount of unreliable and fake information—an “infodemic” [2]—putting lives at risk by prompting the population to try unproven medications in the hope of preventing the disease or finding a “cure” [3]. In this context, telehealth and digital health solutions, including chatbots, emerged as a quick and viable response, acting as a symptom checker in digital triage approaches [1,4,5].

Chatbots are conversational agents that interact with people using a text-based interface or spoken natural language [6]. They are usually deployed through website widgets or instant messaging apps and have been increasingly adopted in several different fields such as finance, commerce, marketing, and fitness [7]. They have only recently started to expand into health care [8]. Their method of communication makes it suitable for a variety of target populations; various health conditions; and a broad range of purposes such as patient triage, clinical decision support, and self-management [9-12].

The potential of chatbots for screening and monitoring COVID-19 was envisioned since the disease outbreak as a strategy not only to disseminate up-to-date and trustworthy information but also to promote healthy social behavior and to support the provision of health care services safely and at scale [13]. For the purpose of pandemic management, chatbots might teach people about social distancing and other prevention measures; clarify doubts about symptoms, treatments, and vaccines; and help screen patients remotely, avoiding unnecessary visits to health care centers that could implicate crowding and taking up valuable time of health care professionals [14].

In this scenario and in view of its far-reaching postpandemic impact, it is critically important to evaluate user experience with this type of technology. Despite the World Health Organization (WHO) recommendation regarding the assessment of user interaction for the adoption of digital technologies in health care, evidence on chatbot assessment in the context of the COVID-19 pandemic and other conditions is still scarce [4,5,15]. This is of utmost importance not only as a way to assess and enhance users’ experiences but also to improve the technology itself, so that it can fulfill its ultimate goal of promoting public health and saving lives even during a scenario of uncertainties from the lack of evidence and ethical risks. In addition, assessment can provide insights for the development of chatbots for other conditions. The better the quality of user experience, the greater the chances of adoption and benefits for most users.

Therefore, this paper sought to evaluate the quality of user interaction with a chatbot developed to respond to the COVID-19 pandemic by a large telehealth service in Brazil to assess users’ overall experiences, including strengths and shortcomings, as reported by participants.

## Methods

### Chatbot Development and Implementation

**Figure 1:**
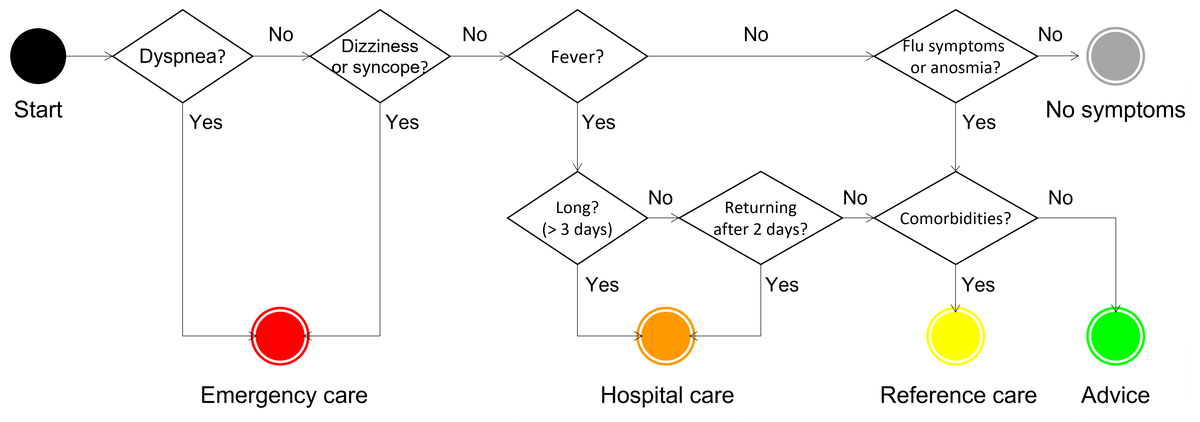
Decision tree for screening suspect cases of COVID-19.

The planning and development of our COVID-19 chatbot were described in detail previously [16,17]. The bot was developed in March 2020 at the beginning of the first wave of the COVID-19 pandemic in Brazil to provide 2 core functionalities. The first was assisting web-based screening of COVID-19 symptom severity based on a decision tree that considered available evidence and recommendations from the Brazilian Ministry of Health [18] and the WHO [19]. This functionality was meant to (1) advise the population whether and when to seek care, with people with no warning signs advised to stay home; and (2) queue patients for teleconsultation, prioritizing those with warning sign severity and comorbidities [20]. Figure 1 shows a flowchart of the stages the user traverses guided by the chatbot questions. Colors are used to screen cases: (1) red (user advised to search for immediate, emergency care); (2) orange (user advised to search for urgent care at the hospital); (3) yellow (user advised to search for care in reference centers); and (4) green (user advised to stay at home unless new warning signs appear).

The second functionality aimed to supply evidence-based information to the population at a time of uncertainty, misinformation, and widespread dissemination of fake news. Misleading information can be created and used unintentionally or intentionally to cause harm (misinformation vs disinformation vs malinformation) [21]. However, there is misleading information from the lack of consistent evidence regarding many aspects of this recent disease, which demanded continuous revision in the scientific basis of the chatbot. This was provided as question and answer (Q&A) based on frequently asked questions in the database at the Telehealth Center of the University Hospital at *Universidade Federal de Minas Gerais* [22]. The questions were initially grouped into 11 topics—general information, transmission, symptoms, advice for suspected cases, treatment, home care, hygiene, lifestyle, mask use, pregnancy, and pet care—and later expanded to include diagnosis. A group of health care professionals at the Telehealth Center selected 85 Q&A pairs based on the best available evidence and following the Brazilian Ministry of Health [18] and WHO [19] recommendations.

**Figure 2:**
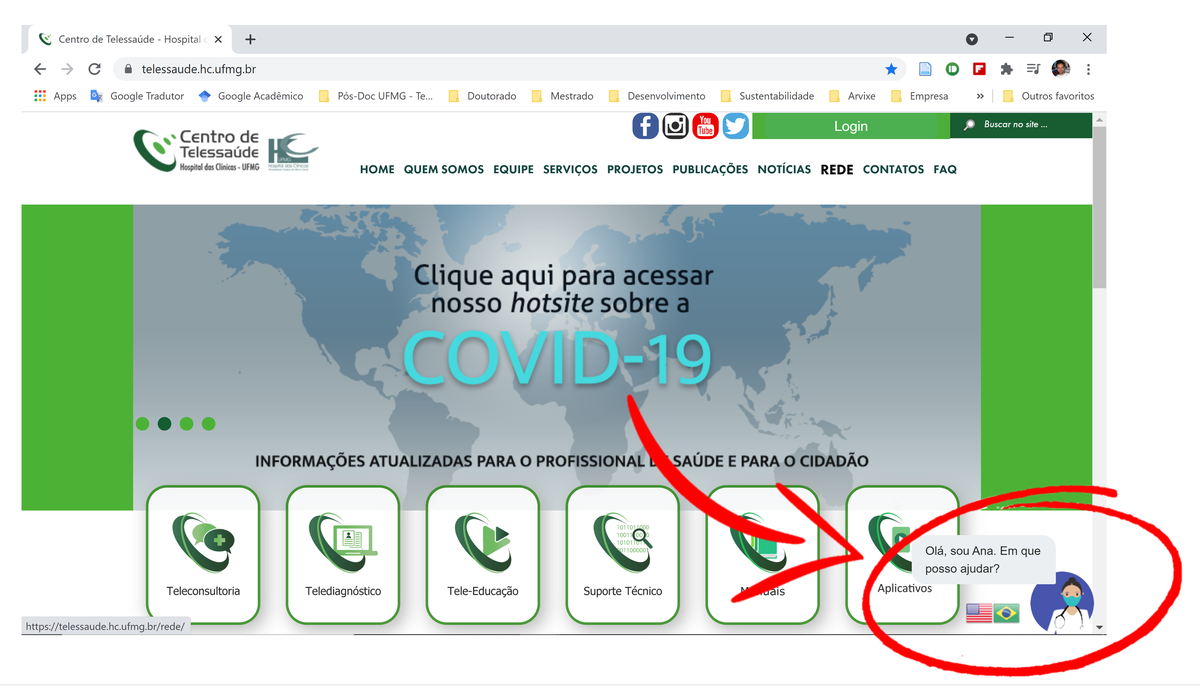
Screenshot of the Telehealth Center website showing our chatbot Ana as a widget at the bottom right of the page.

Our chatbot, having a female identity and the name *Ana*, was developed using BLiP [23]—a proprietary software platform—as a service for the development of conversational agents. The chatbot was available via different channels, namely as an app on WhatsApp (Meta Platforms Inc); as a webchat on the web sites maintained by the Telehealth Center [24] (Figure 2), the city of Teófilo Otoni [25], and the University of São João del Rei in Divinópolis [26]; and as an “embedded” app hosted by Divinópolis municipal health department [20]. A version of the chatbot with a male identity and the name *Pedro* was also made available on the website maintained by Universidade Federal de Minas Gerais for students and personnel to queue for teleconsultations and have access to frequently asked questions. For the purposes of our study, we focused solely on the chatbot *Ana*.

### Study Design

A mixed methods approach was used, and user experience with the chatbot was evaluated through (1) a posttask usability survey administered to a sample of users who resorted to the bot for symptom checking to gather participants’ impressions immediately after concluding their interaction with the bot and (2) an interview with volunteer participants who engaged in simulated interaction with the bot guided by the interviewer. We performed a convergent parallel mixed methods design [27], in which data were collected and analyzed separately, and the results were presented side by side and then related at the end. Both studies address the same macro–research question regarding user experience with the chatbot. The quantitative study is meant to indicate a broad trend, whereas the qualitative study is meant to provide deeper insight into the user experience.

### Ethics Approval

The study protocol was approved by the Brazilian National Commission for Research Ethics (CAAE 35953620.9.0000.5149). Individual informed consent was obtained for all the participants.

### Usability Survey

A brief usability survey was used to assess users’ overall impressions after they had concluded using the chatbot. The survey was intended to evaluate chatbot usability at scale and was administered to all users after concluding their interaction with the chatbot. As they were symptomatic users who were actually quite concerned about their health condition and were not very willing to spend time answering a questionnaire, we opted to use a small set of 4 questions drawing on the classic criteria for usability assessment [28,29]. The questions inquired on 4 usability aspects, namely ease of use, usefulness, satisfaction, and likelihood of recommending the bot to other users. Answers were collected using a 5-point Likert scale, ranging from 1 (worst score) to 5 (best score), representing the strength with which the respondent agreed or disagreed with each question. All users were invited to reply to the survey, but replying was optional, and users could comply and accept the invitation or conclude their chatbot session without answering our survey. From October 2020 to January 2021, 622 complete interactions with the chatbot were recorded. In total, 63 out of 622 users agreed to fill in our usability survey (response rate of 10.1%). Table 1 shows the sociodemographic data of respondents and nonrespondents.

**Table 1:** User profile of recorded interactions^a^.

Users	Age (years), mean (SD)	Women (n=380), n (%)	Men (n=237), n (%)	Not declared (n=5), n (%)	Total (n=622), n (%)
Respondents	36.1 (14.4)	46 (12.1)	17 (7.2)	0 (0)	63 (10.1)
Nonrespondents	34.5 (14.1)	334 (87.9)	220 (92.8)	5 (100)	559 (89.9)
Total	34.7 (14.1)	380 (100)	237 (100)	5 (100)	622 (100)

^a^Information was recorded during interaction as informed by users.

The respondents had a mean age of 36.1 (SD 14.4) years and were predominantly women (46 out of 63 respondents, 73%).

Descriptive statistics assessed the characteristics of the users and responses to the usability questions. To summarize the quantitative variables, we used averages, SDs, medians, minimum and maximum, or IQRs depending on the data distribution. Qualitative variables were presented as absolute values and percentages. Box plots enhanced the visualization of grades assigned by users on each criterion for assessment.

### Qualitative Assessment: Users’ Interviews and Analysis

To tap users’ assessment of the chatbot interface and core functionalities (screening and educational session), we conducted a remote teleconference session with 15 invited volunteer asymptomatic participants having different age, sex, and occupation profiles recruited by the research team. Each participant received a scenario describing a situation that would prompt their interaction with the chatbot. The researcher observed and recorded their interaction. The session was followed by a semistructured interview to gather insights on their experience with the chatbot and their perceptions of the strengths and shortcomings of the bot as reported by them.

The evaluation was conducted through a teleconference system and took place between November 2020 and January 2021 as the second wave of the pandemic started in Brazil. The interviews were transcribed, and a thematic analysis was performed [30].

Among the 15 participants, 53% (n=8) were female, with ages ranging from 18 to 62 (mean 38.1, SD 15.7; median 37; minimum=18, Q1=25, Q3=51, maximum=62) years, and 73% (n=11) had a higher education degree. Out of the participants, 33% (n=5) were engaged in teaching or research at the university, 27% (n=4) were students and 40% (n=6) were regular or self-employed workers. With regard to the device used to interact with the chatbot, 80% (n=12) used a desktop or laptop computer, whereas 20% (n=3) used a smartphone. The participants’ data are detailed in Table 2.

In the evaluation session, the participants received a scenario describing a situation that would prompt their interaction with the chatbot. A set of 10 different scenarios were prepared to cover different chatbot interactive paths in the screening functionality, from severe to light symptoms, with and without comorbidities (Multimedia Appendix 1). Participants were designated to scenarios according to their actual profiles to make the interaction as realistic as possible. Sample scenarios included an adult woman in her 30s being assigned a scenario of a pregnant woman, a participant in their 60s being assigned a scenario of a person with some comorbidity, among others. Similarly, each scenario included 3 topics to assess the educational functionality of the chatbot, 2 of them being preassigned topics, and a third one free for the participant to choose. Most sessions lasted between 30 minutes and 1 hour. During the sessions, the participants interacted with the chatbot while the researcher observed and recorded their interactions. Afterward, they were interviewed about their experience with the chatbot (the interview script used is available in Multimedia Appendix 2). The evaluation was conducted through a teleconference system chosen by the participant, in an individual session and in Portuguese (participants’ mother tongue), which took place between November 2020 and January 2021.

**Table 2:** Profile of participants taking part in the semistructured interview.

Participant	Age (years)	Sex	Education	Occupation	Device used
P01	37	Male	Graduate degree^a^	University lecturer	Desktop or laptop computer
P02	48	Female	Graduate degree^a^	University lecturer	Desktop or laptop computer
P03	25	Male	Graduate degree^a^	Attorney	Desktop or laptop computer
P04	40	Male	Graduate degree^a^	IT or user experience designer	Smartphone
P05	25	Female	Bachelor’s degree in linguistics	Student pursuing a master’s degree	Desktop or laptop computer
P06	58	Female	Bachelor’s degree in nutrition science	Credentialed dietitian and undergraduate student in psychology	Smartphone
P07	27	Female	Bachelor’s degree in veterinary studies	Undergraduate student in linguistics	Desktop or laptop computer
P08	52	Female	Graduate degree^a^	Lecturer	Desktop or laptop computer
P09	33	Male	Graduate degree^a^	Sociologist	Desktop or laptop computer
P10	50	Female	Graduate degree^a^	Psychologist	Desktop or laptop computer
P11	20	Female	High school degree	Undergraduate student in psychology	Desktop or laptop computer
P12	18	Female	High school degree	Student	Desktop or laptop computer
P13	59	Male	Bachelor’s degree in computer science	IT analyst	Desktop or laptop computer
P14	18	Male	High school degree	Undergraduate student	Desktop or laptop computer
P15	62	Male	High school degree	Insurance broker	Smartphone

^a^Master’s or doctoral degree.

The interviews were recorded and included screen recordings of participants’ interactions with the chatbot. The interviews were transcribed by the research team. Thematic analysis [30] of the interview transcripts was carried out to find recurrent themes in participants’ interviews that could be matched to the research questions guiding our study as follows:

1. What are the strengths and shortcomings of our bot as perceived by users?
2. What particular insights can be drawn from our study to inform prospective chatbot design?

Our thematic analysis was conducted in an inductive way, that is, a bottom-up approach, where the analysis is not driven by a preexisting framework or theory, but the researchers search for codes and themes in a data-driven way [30]. This approach is applicable for qualitative analysis of interview data [31] and is more suitable for broad rather than specific research questions, as was our case [30].

We applied triangulation as a typical strategy to improve the quality and reliability of our qualitative results [32]. In particular, the data were analyzed by multiple researchers (investigator triangulation [33,34]) and the outcome of their analysis was discussed until consensus was reached. The transcripts were coded by 2 senior and 2 junior researchers, with a set of at least 5 transcripts being assigned to each one for analysis and coding. Thus, every interview was analyzed by at least 2 different researchers. Interviews were recorded and analyzed in the qualitative data analysis using Miner Lite (Provalis Research) software [35], which is adequate for qualitative analysis. Finally, the codes were presented to peers, refined, and organized as per this report in discussions with other senior researchers from the team.

## Results

### Usability Questionnaire

**Figure 3:**
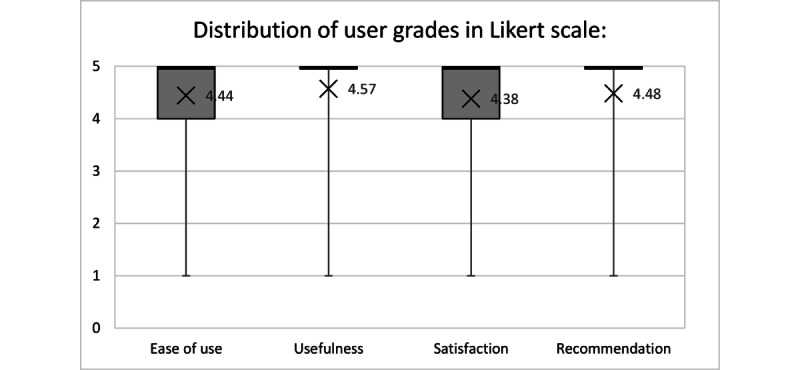
Box plot for grades assigned by users on each criterion for assessment.

Table 3 shows the questions asked and the number of users who assigned each grade to each criterion. The bot obtained high grades on all evaluation criteria. App usefulness obtained the highest mean (4.57), whereas satisfaction attained the lowest mean (4.38). Figure 3 shows a box plot of the grades assigned by users as per quartile distribution in Table 4, clearly indicating predominance of grades 4 and 5 with few outliers.

**Table 3:** Grades assigned by users to each question on chatbot usability within a scale of 1 (lowest grade) to 5 (highest grade; n=63).

Question	Grade, n					Total, n	Values, mean (SD)
	1	2	3	4	5		
1. Was this app easy to use?	4	3	1	8	47	63	4.44 (1.16)
2. Was this app useful to you?	2	0	6	6	47	61	4.57 (0.92)
3. Was this app satisfactory to use?	4	1	6	6	43	60	4.38 (1.17)
4. Would you recommend this app to other people?	4	2	2	5	46	59	4.47 (1.16)

**Table 4:** Quartile distribution of grades assigned by users to each question on chatbot usability.

	Ease of use, n	Usefulness, n	Satisfaction, n	Recommendation, n
Minimum value	1	1	1	1
Quartile 1 (25%)	4	5	4	5
Quartile 2 (50%): median	5	5	5	5
Quartile 3 (75%)	5	5	5	5
Maximum value	5	5	5	5

### Qualitative Assessment

Initially, excerpts were annotated with the following tags: positive feedback (aspects reported as positive by the participants regarding interaction, interface, and content of the chatbot); negative feedback (points considered negative by the participants pertaining to interaction, interface, and content of the chatbot); and neutral (comments that did not qualify as either ostensibly positive or negative). Different themes emerged in each broad category (positive or negative).

As a following step, we analyzed each of the themes and organized them based on whether they were related to the *usability* of the chatbot or to the *health support* it offered. Themes associated with the *usability* of the chatbot pertained to issues related to the system’s interface, the users’ perspective of the effectiveness and efficiency of the proposed functionalities, and users’ perceptions and responses to the use of the system. *Health support* included all themes that addressed aspects of how the chatbot achieved its goal to offer support regarding COVID-19 screening and education to users.

Our classification allowed us to reveal and point to problems related to different sources in our COVID-19 chatbot—design decisions of the technology itself and how it supports users’ needs for health information in the context of COVID-19 screening and education. Next, we present the results of our analysis and describe each identified theme. We present both the positive and negative aspects that emerged from our analysis. Nonetheless, we examined the negative aspects in more detail, as they point to the aspects that still need to be improved and dealt with in health chatbots.

### Positive Feedback

On the basis of our analysis of the positive comments from participants, 6 different themes emerged—3 related to the usability of the chatbot and the other 3 pertaining to health support.

Regarding the chatbot’s usability (Table 5), participants in general found the interface esthetically pleasing and with good usability (C1). They reported an overall positive experience with the chatbot, mostly because of its ease of use (C2). Finally, some participants showed a reasonable level of understanding about the chatbot’s underlying logic, which is positive in the sense that the interaction improves as the user understands how the technology works (C3).

As for the themes associated with health support (Table 6), participants valued the fact that the screening process was simple and straight to the point, helping users understand the action they should take (C4). Furthermore, they found that there was a broad range of topics in the Q&As, including content related to fake news that had been circulating at the time, and considered the answers concise and easy to understand, a very frequent comment in their interviews (C5). Finally, participants found that the chatbot was useful and valuable, especially considering the circumstances they were living in at the time—it was trustworthy and allowed them to obtain reliable information without the risk of getting infected (C6).

**Table 5:** Codes, number of occurrences, and examples of positive feedback regarding usability (we translated excerpts into English).

Code	Description	Occurrences, n	Examples
C1. Chatbot interface design and functionalities	Comments on chatbot graphic interface design, including font-size and text display on screen, chatbot esthetics, and use of button-limited options	4	“Well, I liked having a menu with numbers and that you can just type a number, because I think it makes it much faster.” [P05] “Spacing in the text display is adequate and the text fits almost all the screen, right? It’s not so small that we have to strain our eyes to read it, and it is also easy to scroll the screen up and down to be able to go back to check something.” [P13]
C2. Positive user experience	General positive comments on overall experience of interacting with the chatbot, for example, ease of use	26	“I thought it (the experience) was nice, the bot was very easy to use and you get clear instructions about what you have to do, there is no way you can get lost.” [P12] “It is very good that it is responding very fast. It doesn't have that, you know, delay to answer.” [P03]
C3. Understanding chatbot underlying rationale	Comments on user perception and understanding of rationale behind the chatbot operation	3	“There is an interesting logic underlying the bot. It looks like a flowchart, right? You have a yes or no question, then depending on the answer, you go to another group of questions...” [P01] “...I noticed that depending on the information I submit about my symptoms, it will give me a direction, it will direct me to where to go, isn’t it?” [P15]

**Table 6:** Codes, number of occurrences, and examples of positive feedback regarding health support (we translated excerpts into English).

Code	Description	Occurrences, n	Examples
C4. Patient screening session—process and guidelines	Considerations about directions given, color system used in the triage phase, and chatbot guidance during the screening session	18	“I thought the bot was very cautious (in its assessment), because I reported having symptoms considered severe, right? So the bot immediately told me to seek help as soon as possible.” [P13] “I thought the guidelines were very clear: options about what you needed to do, if you had any symptoms...like fever...and also questions about you belonging to a risk group, having a risk condition, right? Which could mean a more severe Covid case.” [P01]
C5. Question and answer session—range of topics and trustworthiness of information provided	Number and content of answers considered satisfactory as well as effective in expanding knowledge about disease	67	“The content, the number of questions, topics...I was positively impressed. If you had asked me how many general topics I could think of, I wouldn't have thought of 12 at all. You know? That’s what I liked the most, that there’s also a lot of information to be explored.” [P03] “I thought it [the experience] was very, very positive. I really liked the content ... it is very reliable. These are true guidelines, everything is correct.” [P06]
C6. Reported advantages of chatbot use during COVID-19 pandemic	Motivations and advantages of using a chatbot during the COVID-19 pandemic	18	“I would use the bot and recommend it to friends and acquaintances who might want to have some reliable information, because there is plenty of fake news about Covid. And even though it is a robot, you do get a reliable answer; there is no fake information. The bot provides very straightforward answers that point to what should be done.” [P15] “Here, where I live, this chatbot would be my first option to seek advice, for sure, because I don’t have many options...to get such guidance, to avoid going somewhere where I might get infected, or to speed up my recovery from the disease.” [P08]

### Negative Feedback

On the negative side, although we obtained approximately the same number of excerpts as in positive feedback, our analysis led to a larger set of categories, 10 of them related to usability and 5 related to health support.

Regarding usability (Table 7), different types of problems emerged, from technical problems to interaction and interface design problems to problems with the expectation of better communicative capability (which would require artificial intelligence [AI] support). Technical problems were reported by participants who faced difficulties when sharing their location with the chatbot (C7), and sometimes, the app became slow or unresponsive in their mobile phones (C8).

Although participants considered their overall experience with the chatbot to be good, they commented on many issues that could improve the interaction if solved. Regarding the flow of the conversation, some participants had difficulties when trying to go back after typing a wrong option (eg, C9). Some participants complained about the menu being displayed too quickly and hindering their ability to read the chatbot’s (previous) response (C10). Another problem related to conversation flow was observed when participants did not understand how their interaction with the chatbot evolved. For instance, a participant missed the cue indicating that the chatbot was answering and did not wait for it to respond before sending another message (C11).

The lack of graphical interactive resources (eg, clickable menu options) was also an issue for some participants (C12). Another problem we observed in some sessions was participants not knowing how to start the conversation with the chatbot and asking the interviewer for guidance owing to absence of basic initial directions (C13). Some comments about the chatbot language were also pointed out by participants who thought it may not be adequate for users with lower levels of literacy (ie, they may not be able to understand it), which indeed can be an issue in Brazil (C14).

**Table 7:** Codes, number of occurrences, and examples of negative feedback regarding usability (see Multimedia Appendix 3 for more examples).

Code	Description	Occurrences, n	Examples
C7. Difficulty in sharing location	Participant unable to share device GPS location when requested by chatbot	10	“What should I do here about this location?” [P12]
C8. Technical problem in mobile app or phone	Chatbot stops responding or gets slow; interaction is interrupted	4	(P10 started using the chatbot on her mobile phone, when the bot stopped responding for the third time during interaction) Interviewer: “Right, it should have answered you already, I really don’t know what’s happening...”
C9. Need for an option to go back and make a different choice during interaction	Participant needs to start over and repeat entire session, as the chatbot does not have an option for backtracking or choosing a different path during conversation	2	(P04 inattentively selected the Q&A functionality and had to start over to select the screening one) Interviewer: We can start with Q&A, or you can start over so you can select screening. P04: I’d rather start over. Let’s go.
C10. User choice repeatedly prompted by option menu and at high pace	Participant complaint about being prompted to make a choice in option menu and finding it too fast to be able to read the whole answer provided by the bot	6	“What I didn’t like, but I don’t know if it can be improved, is that the menu prompts you to select an option all the time. This is my feeling about the way the bot operates and not a negative feature of the chatbot.” [P14]
C11. Conversation flow management	Participant does not succeed in keeping the conversation flowing with the chatbot owing to unperceived feedback or lack of it from the chatbot (eg, turn taking management)	4	“It was difficult until I understood that I had to wait for the bot to answer to keep the conversation flowing.” [P15]
C12. Better interface resources	Additional features in chatbot interface to enhance interaction	12	“Maybe answers should be formatted differently because then you would clearly distinguish question and answer.” [P10]
C13. Insufficient directions on how to interact with the chatbot	Participant requesting directions or help from the interviewer	22	“To be honest, at first I found it difficult to understand what I had to do: I tried to click on the number of the option I wanted to select. Then I realized that I needed to type the number. So that was my first problem using the bot.” [P15]
C14. Chatbot language need to be adapted to meet different user profiles	Language used by the chatbot needs to be adapted to be understood by user with low-literacy level	2	“When the app starts you could ask the user’s level of education, and if a user reports a low level, the bot may switch to answers that are more adapted to the user’s literacy level.” [P01]
C15. Chatbot fails to understand unexpected user responses	Chatbot does not successfully process information entered by the participant	11	Participant 10 enters “50 years old” in the age field, and the chatbot asks her to enter only a numerical value. The participant then types “50,” which is successfully processed by the chatbot and interaction is resumed.
C16. Participants expectations exceed chatbot’s actual communicative ability	Participant tries to interact in a way not supported by the chatbot, for example, by trying to speak to the chatbot by voice	5	P06: So I should type that I would like to know more about pregnancy? [Participant starts typing “I want to know more about pregnancy” Interviewer: Actually, each number is a shortcut, you don’t have to type everything.

Finally, we also observed some interaction problems related to our chatbot technology limitations. Some participants entered unexpected text inputs into the chatbot that it was not prepared to handle (C15). Similarly, others tried to interact with the chatbot by typing or even speaking in natural language (C16). Both issues could be addressed by applying better support for natural language processing and understanding using AI, which was already commonly found in several conversational systems at the time.

Regarding health support (Table 8), participants commented on some outdated or missing information they noticed in some answers (C17 and C18). It is worth pointing out that interviews took place at the end of 2020 when there was still much to be learned about COVID-19. Furthermore, this was around the time when the vaccine was underway and the chatbot did not have any information about it yet. In some cases, participants reported dismay with the briefness of the clinical evaluation during the screening session (a participant, for instance, expected a more detailed and thorough evaluation of her symptoms before the chatbot gave her instructions) and the lack of mechanisms to mitigate responses for severe symptoms (C19). Finally, the last themes have to do with the need, mentioned by some participants, for more practical and situated guidance or information both in the screening section (C20) and in the Q&A section (C21).

As is the case with any qualitative analysis, numeric information should be interpreted cautiously and is presented here for the sake of transparency. It should be noted that the number of tagged occurrences of a code is not a general indicator of relevance or importance, because our analysis was not based on frequency or other statistical metrics. Thus, this information is not meaningful to discuss codes’ validity [31] and was included as an index of the overall analysis process, not to indicate any validation of the analysis. Table 9 shows that the number of negative codes is greater than the number of positive codes. This is expected because we analyzed the negative aspects more thoroughly, as stated earlier, leading to individually less frequent and more fine-grained negative codes. In the category level, frequency of codes can be an approximate indicator of the distribution of positive and negative aspects. In Table 9, we can see that 54.8% (136/248) of the excerpts were identified as positive, and the remaining 45.2% (112/248) were identified as negative.

**Table 8:** Codes, number of occurrences, and examples of positive feedback regarding health support (see Multimedia Appendix 4 for more examples).

Code	Description	Occurrences, n	Examples
C17. Outdated information or answer	Participants noticed some outdated information or questioned whether the information presented in the Q&A^a^ session was updated	4	“How often is the FAQ updated? For instance, whether there’s a vaccine or not...Because that ensures credibility, right?...Because sometimes people notice that the information is a little outdated.” [P13]
C18. Missing information or explanation	Participants suggested a topic in the Q&A session that should be included or further explained	5	“I think there could be some explanation on IgG tests [after reading about IgG tests on one of chatbot’s answers]. Because many people have been talking about it and they don't know what that is.” [P11]
C19. Unfulfilled expectations during the screening session	Participants mentioned interesting insights and broken expectations during the screening process	13	“...maybe the person wanted a little bit more information before the chatbot said: ‘Go to the hospital’ [laughs], you know? ‘Go to the hospital because this is a serious symptom.’ Maybe something in the sense of reassuring the person, like...‘look, these are symptoms that can be considered’...the direction could be modalized, so as not to scare the person.” [P02]
C20. Need or demand for actionable orientation during the screening session	Participants expected to receive more practical instructions at the end of the screening session	5	“I think there should be something more direct to guide the next step. What am I supposed to do? The bot gave me some explanations about Covid, about my condition, but it didn’t tell me where to go. Given that the person in my scenario is in a risk group, as she's pregnant, I thought people would need to know about this.” [P06]
C21. Demand for situation-oriented answers to questions	Participants expected to find answers that could be more directly applied to a particular situation in the question and answer session	7	“If you have traveled, is there a test that allows you to go to your relative's house without having to worry? Or if you actually have to isolate yourself and wait 3 days to see if you won't have anything after leaving the airport?” [P09]

^a^Q&A: question and answer.

**Table 9:** Summary of code categories.

Category		Codes, n	Tagged occurrences (n=248), n (%)
**Positive**		6	136 (54.8)
	User experience	3	33 (13.3)
	Health support	3	103 (41.5)
**Negative**		15	112 (45.2)
	User experience	10	78 (31.5)
	Health support	5	34 (13.7)
Total		21	248 (100)

## Discussion

### Overall Findings

The WHO guidelines point out that considering the potential impact that interface and interaction issues have on health care services and even on clinical practice [15], it is essential to evaluate user experience in health care systems. Despite the increased use of chatbots in a range of fields, this form of technology has yet to be robustly assessed, and the literature regarding these conversational agents’ formats, focusing on their acceptability, safety, and effectiveness, is still incipient [7]. Moreover, the lack of standardization and paucity of objective measures make it difficult to compare the performance of health chatbots [36].

In this paper, we present users’ evaluations of a chatbot developed specifically for screening cases and supplying information regarding COVID-19. We performed a brief, quantitative assessment with actual chatbot users and an in-depth evaluation with participants through simulated scenarios (volunteers who were asymptomatic and engaged in chatbot interactions as guided by the interviewer).

Although our quantitative analysis indicated that overall users were satisfied with the chatbot, our qualitative analysis allowed us to identify participants’ perspectives of positive and negative aspects regarding usability and health support, as described in sections *Positive Feedback* and *Negative Feedback*. The positive comments from the qualitative study corroborate the quantitative results we found, as positive comments represented approximately 55% of all comments, and the most frequent codes emphasized an overall positive experience (C1) and the usefulness of the provided health support during the pandemic context (C4-C6). At the same time, the negative comments in the qualitative study are not in conflict with the overall positive experience from the quantitative study. All volunteers from the qualitative study reported having an overall positive experience with our chatbot during the interviews. The negative comments should be interpreted as opportunities for improvement that did not compromise the overall experience. In the subsequent sections, we discuss some of the main issues based on our analysis.

### Updated Chatbot Information

The results indicate that the pandemic context created specific circumstances that led participants to assign value to having a chatbot available—fake news dissemination about COVID-19 and the disease’s high transmission rate. This means participants welcomed the possibility of having access to reliable information at a time when plenty of fake news about COVID-19 was circulating in Brazil, presumably connected to political interests and governmental sources as well as misinformation and infoxication from inappropriate scientific papers [37]. Furthermore, knowledge about COVID-19 was rapidly evolving, and the population was seeking sources of trustworthy information. Participants also felt that obtaining directions as to how to proceed in case of symptoms without having to be exposed to chances of getting infected by the virus was a positive factor. On the other hand, because information evolved so quickly, participants noticed that information provided by the chatbot was not fully up to date (eg, about vaccines, which were underway). This was perceived as a negative impact that could undermine the reliability assigned to the chatbot and points to the challenge of the need for constant information updating in conversational agents. This includes deciding which pieces of new information are relevant to be included and how to best translate new scientific evidence for the lay population, a similar challenge faced by decision support systems in general [38]. As previously stated, developing a high-quality COVID-19 chatbot is critical but not enough for widespread adoption. It is fundamental to demonstrate and emphasize that chatbots are able to deliver the same quality service as human agents [39].

### Universal Usability

Another aspect that emerged in our analysis, which is very relevant to the Brazilian context and may also be relevant to other resource-limited countries, is the need for universal usability [40], that is, to provide access to technology to all citizens. In the case of our chatbot, issues related to access quality were observed by the interviewer or directly reported by the participants in our evaluation. Some participants used their own cell phones for assessing the chatbot. However, in one case, the participant had technical problems that were not software bugs strictly speaking but seemed to be associated with users’ device limitations related to the operational system and to hardware resources. Although currently there are more smartphones in use in the country than citizens [41], owing to the inequalities in our country, the chatbot may not be universally accessible through all smartphone models in use. Furthermore, one participant specifically raised the issue of the educational level of other Brazilian citizens and pointed out the need to adapt the chatbot’s language to a larger variety of user profiles. These results corroborate not only the need to assess user experience of health care technology in general but also issues brought about by local conditions of technology use in a country or region.

Beside quality of access to technology, literary and accessibility issues are also issues to be tackled in chatbot development. Srivastava [42] reviewed gaps found in using chatbots during COVID-19, and one of them was “inaccessible information,” that is, most of the chatbots created assumed that the users were literate, experienced with digital technology, and did not have any disabilities. These assumptions prevented a considerable part of the society from benefiting from chatbot technology. In the case of our chatbot Ana, our team performed several updates in the chatbot language, aiming at making language more accessible to less-literate users and enhancing user experience.

### Expected Communicative Abilities

Analyzing the negative aspects of interacting with the chatbot, we noticed that most of them (6 out of 10—C9, C10, C11, C13, C15, and C16) were related to the conversational paradigm adopted in such technologies. Participants reported on interactive breakdowns (ie, problems they had as they interacted with the system) that were generated by many different causes—from not knowing exactly how to interact with it (C13) to expecting too much of the bot’s communicative abilities (C16). Although these challenges are mainly related to chatbots in general [43-47] and not only in the health domain, they emerged as hindering users’ interaction with the system and impacted (negatively) their experience with it, which could lead them to not fully embrace or adopt the technology.

### Complete Health Care Information

It is important to understand the negative aspects related to the health support offered by the chatbot, as this is the technology’s main goal. Out of the 5 themes describing these negative aspects, 2 of them, as mentioned, were related to the need to keep information updated and complete when knowledge of the disease was continuously evolving during the beginning of the pandemic. A third one was the system not fulfilling users’ expectations regarding a more thorough clinical evaluation or more careful instructions to patients. The decision-making framework of a chatbot is crucial to address this issue. Models based on user-initiated solutions are usually easier to deploy; however, this type of solution may be insufficient in some scenarios and may lead to situations in which a high-risk person or a person with issues regarding specific conditions or contexts would rather seek an in-person assessment in a health care facility, because they did not feel safe or could not follow the recommendations. On the other hand, models based on provider-initiated solutions allow providers to “close the loop” and properly address more specific conditions [11].

### Contextual Information and Adaptive Ability

Finally, the last 2 themes (C20 and C21) point to the expectation that some users highlighted of having more practical orientation and situation-oriented information. These kinds of features would demand more sophisticated technologies that might be able to handle contextual information from users to identify contextual needs and adaptively respond to them. To achieve this type of goal, we would need at least a richer data set comprising a reasonable set of different situations and Q&A pairs and more sophisticated technologies able to detect and handle users’ contexts appropriately. Context could be inferred from isolated conversations but would probably be better constructed by technologies that combine external variables (historical data, location, etc) such as search engines and advertisement technologies. We believe that adaptive AI capabilities such as recommender systems can be included in the chatbot to provide more specific instructions that would take into consideration the users’ specific condition (eg, comorbidities) or context (eg, location) in the answers and piece of advice given.

An adaptive approach can also be used as a strategy to address users’ diversity in skills and preferences. We observed conflicts between the participants’ comments and opinions, such as C1 contradicting C9, C10, and C12 and C3 contradicting C13. As mentioned before, most participants in the qualitative study had an overall positive experience with our chatbot, and negative comments should be interpreted as opportunities for improvement and not as a conflict of results. However, the participants were bothered by our chatbot’s problems when interacting with the system in different ways, depending on their profile, background, patience at the time, etc. Fulfilling the goals and needs of a large diversity of users with different profiles, backgrounds, and preferences is also a goal at the core of the universal usability principle [40] and one of the major interaction design challenges. We believe that adaptive chatbots should be investigated as a promising technology to help in this regard.

### Directions for Future Research

The construction and deployment of a chatbot for COVID-19 is a dynamic project that demands collaboration among multiple disciplines such as health professionals, linguists, technology designers, and developers [16]. The results of our qualitative analysis and discussions provide directions for multidisciplinary teams to approach projects of prospective bots and are expected to help organize the problem space of regarding interaction decisions and issues to help understand users’ needs and expectations in such endeavors.

### Limitations

Although our qualitative evaluation of the chatbot included a small sample of 15 participants, there was a distribution of gender (7 female and 8 male participants) and age, varying from 18 to 62 years. Nonetheless, as the assessment was performed during the pandemic through teleconference, it required participants who had access to computers and good internet bandwidth. Thus, it does not represent the variety of educational or economic groups in Brazil. In the future, our goal is to broaden our evaluation to include other groups of our population who represent potential users of the system. Moreover, we did not investigate the perceptions of physicians, nurses, and caregivers regarding the use of this COVID-19 chatbot, including their benefits, challenges, and risks to patients.

This qualitative study was designed to allow the collection of rich, in-depth data containing participants’ thoughts and insights about their experience of using our chatbot. At that time, using chatbots for health purposes was not common in Brazil, and the interviewer’s clarifications during sessions were given to participants to unblock them from dead ends, thus enabling us to collect more rich and useful data. All such cases were annotated and considered in the analysis.

Our quantitative assessment of our COVID-19 chatbot was evaluated by 10.1% (63/622) of users who chose to participate in the evaluation process. Further analysis is needed to test their statistical significance. As the system continues to be used, we expect more users to willingly participate and more data to be collected regarding their attitudes toward the system.

### Conclusions

This study evaluated the quality of user experience with a chatbot designed in response to the COVID-19 pandemic by a large telehealth service in Brazil through an analysis of usability with real users and an exploration of strengths and shortcomings of the chatbot, as revealed in reports by participants in simulated scenarios. Our results indicate that overall, users had a positive experience with the chatbot and found the health support relevant. Nonetheless, the qualitative evaluation of the chatbot indicated challenges and directions to be pursued in improving not only our COVID-19 chatbot but also health chatbots in general.

## Appendix

Multimedia Appendix 1 Scenarios for in-depth chatbot evaluation (asymptomatic users).

Multimedia Appendix 2 Script for the Interview conducted with asymptomatic (healthy) participants following their interaction with the chatbot.

Multimedia Appendix 3 Complete version of Table 7.

Multimedia Appendix 4 Complete version of Table 8.

## Abbreviations

AI: artificial intelligence
Q&A: question and answer
RCT: randomized controlled trial
WHO: World Health Organization

## Data Availability

Data are available on reasonable request.
