# Supplementary material for: Evaluating User Experience With a Chatbot Designed as a Public Health Response to the COVID-19 Pandemic in Brazil: Mixed Methods Study
Source: JMIR Hum Factors. 2023 Apr 3;10:e43135. doi: 10.2196/43135 (PMC10131797; doi:10.2196/43135)
Supplement: Multimedia Appendix 1 [file humanfactors_v10i1e43135_app1.pdf]

Multimedia Appendix 1 - Scenarios for In-Depth Chatbot Evaluation (asymptomatic users)

The Table below deploys scenarios that were assigned to participants based on their characteristics. Each scenario will lead the chatbot to screen the user in terms of risk and referral. Using the scenario assigned to them, each participant was asked to:

- Use the chatbot by selecting the option "I think I'm sick" to prompt the screening process until getting guidance on their condition .
- Use the chatbot by selecting the option "read updated information" to query for updated information with the option on at least 3 topics in the Q&A.

# Scenario	Patient characteristics	Symptoms	Clinical conditions	Screening	Description
1	female, 25-40 years old	fever for 1 day	pregnant	Yellow	You have had a fever for a day. Usually in such a situation, you rest or take some over-the-counter medicine to reduce the fever. However, as there is a Covid epidemic and you are pregnant, you want to know what you should do in the present situation. You want to get reliable recommendations about whether or not to see a doctor, and what other symptoms are that could indicate that you have Covid.<br><br>You have recently read a news article about a chatbot developed at UFMG that is intended to provide information on Covid. So you have found it and you are going to interact with it to see if it helps you get the information you want.<br><br>After the screening process, you will use the app to query for information and ask questions about Covid on topics 11, 7, and any other topic(s) of your choice.

# Scenario	Patient characteristics	Symptoms	Clinical conditions	Screening	Description
2	male, 50-60 years old	stuffy nose, loss of smell or taste	high blood pressure<br>diabetes	Yellow	Your father is 60 years old and for some days has had a stuffy nose. Now he says he can't smell or taste anything. As you have heard on television that this might be a symptom of Covid, you are very worried especially because your father has high blood pressure and diabetes, which places him within one of the risk groups. So you want to know if you should take your father to an emergency care facility, and in case it is recommended for him to stay at home, how you should monitor your father's condition.<br><br>You have recently read a news article about a chatbot developed at UFMG that is intended to provide information on Covid. So you have found it and you are going to interact with it to see if it helps you get the information you want.<br><br>After the screening process, you will use the app to query for information and ask questions about Covid on topics 1, 10 and any other topic(s) of your choice.
3	female, 18-25 years old.	shortness of breath	healthy condition prior to present symptoms	Red	You are healthy and have observed Covid recommendations in the news and on television. You have refrained from leaving your house, but occasionally you have gone shopping and ordered deliveries. Today you have started to feel unwell and have experienced shortness of breath. Since you

# Scenario	Patient characteristics	Symptoms	Clinical conditions	Screening	Description
					heard on television that this could be a symptom of Covid, you are worried. It is late at night. You want to know if you need to see a doctor immediately or if you can wait until tomorrow to go to a healthcare facility.<br><br>You have recently read a news article about a chatbot developed at UFMG that is intended to provide information on Covid. So you have found it and you are going to interact with it to see if it helps you get the information you want.<br><br>After the screening process, you will use the app to query for information and ask questions about Covid on topics 3, 9 and any other topic(s) of your choice.
4	male, 30-50 years old	feeling about to faint	high blood pressure	Red	You are 45 years old and have not been feeling well for a couple of days. Now you are feeling as if you are about to faint. Since you have heard on television that this could be a symptom of Covid, you are very worried, especially because you have had to leave your home several times during this period for work-related reasons. So you want to know if you should go to a hospital or if you can go to the nearest health center.<br><br>You have recently read a news article about a chatbot developed at UFMG that is intended to provide information on Covid. So you have

# Scenario	Patient characteristics	Symptoms	Clinical conditions	Screening	Description
					found it and you are going to interact with it to see if it helps you get the information you want.<br><br>After the screening process, you will use the app to query for information and ask questions about Covid on topics 4, 6 and any other topic(s) of your choice.
5	female, 20-30 years old	stuffy nose and fever for past 4 days	healthy condition prior to present symptoms	Orange	You are healthy and have been taking the necessary measures with regard to Covid as you have heard it recommended on television and in the news. You have refrained from leaving your home, but you do occasionally go shopping, order deliveries, and have occasionally attended social gatherings. About 4 days ago, you came down with a cold and a stuffy nose. Since you have been having a fever and have heard on television that this could be a symptom of Covid, you have become very concerned. So you want to know whether it is recommended to see a doctor, and in case you are advised to stay home, what to do to take care of yourself.<br><br>You have recently read a news article about a chatbot developed at UFMG that is intended to provide information on Covid. So you have found it and you are going to interact with it

# Scenario	Patient characteristics	Symptoms	Clinical conditions	Screening	Description
					to see if it helps you get the information you want.<br><br>After the screening process, you will use the app to query for information and ask questions about Covid on topics 2, 5 and any other topic(s) of your choice.
6	female, 50-65 years old	fever for 3 days, gone for 2 days and resumed now	chronic kidney disease	Orange	You have had a fever a few days ago, got better for a couple of days, and now have a fever again. Since you heard on television that this could be a symptom of Covid, you are very concerned especially because you have chronic kidney disease and need to undergo frequent hemodialysis, which can place you within a risk group. So you want to know whether it is advisable for you to see a doctor, and in case you are advised to stay at home, how to monitor your condition and deal with the need to leave your house for hemodialysis..<br><br>You have recently read a news article about a chatbot developed at UFMG that is intended to provide information on Covid. So you have found it and you are going to interact with it to see if it helps you get the information you want.<br><br>After the screening process, you will use the app to query for information and ask

# Scenario	Patient characteristics	Symptoms	Clinical conditions	Screening	Description
					questions about Covid on topics 5, 6 and any other topic(s) of your choice.
7	male, 60-70 years old	stuffy nose, sneeze and dry cough	diabetes	Yellow	You have been having a cold, a stuffy nose, sneezing, and a dry cough. Since you have heard on television that this can be either a common cold or covid symptoms, you are worried, especially because you have diabetes, which places you within a risk group. So you want to know whether it is advisable for you to see a doctor, and in case you are advised to stay at home, how to monitor your condition.<br><br>You have recently read a news article about a chatbot developed at UFMG that is intended to provide information on Covid. So you have found it and you are going to interact with it to see if it helps you get the information you want.<br><br>After the screening process, you will use the app to query for information and ask questions about Covid on topics 5, 8 and any other topic(s) of your choice.
8	male, 18-25 years old	none	healthy condition	Asymptomatic	You are healthy and have been taking the necessary measures with regard to Covid as you have heard it recommended on television and in the news. You have refrained from leaving your home, but you do occasionally go shopping and order deliveries. You have not felt sick at all,very

# Scenario	Patient characteristics	Symptoms	Clinical conditions	Screening	Description
					but live with your family and are concerned that you might have caught Covid. You want to know whether you have caught the disease and what you should do to take care of yourself and your family.<br><br>You have recently read a news article about a chatbot developed at UFMG that is intended to provide information on Covid. So you have found it and you are going to interact with it to see if it helps you get the information you want.<br><br>After the screening process, you will use the app to query for information and ask questions about Covid on topics 1, 12 and any other topic(s) of your choice.
9	male, 30-45 years old	catarrh cough and fever for past two days	cardiac condition	Yellow	You came down with a bad cold, with cough and catarrh and fever about 2 days ago. Since you have heard on television that this can be either a common cold or a covid symptom, you are worried, especially because you have had to leave your house occasionally due to work demands and you have a cardiac condition. So you want to know whether it is advisable for you to see a doctor, and in case you are advised to stay at home, how to take care of your and your family's health.

# Scenario	Patient characteristics	Symptoms	Clinical conditions	Screening	Description
					You have recently read a news article about a chatbot developed at UFMG that is intended to provide information on Covid. So you have found it and you are going to interact with it to see if it helps you get the information you want.<br><br>After the screening process, you will use the app to query for information and ask questions about Covid on topics 2, 6 and any other topic(s) of your choice.
10	female, 20-30 years old	stuffy nose and fever for past day	healthy prior to present symptoms	Green	You are healthy and have been following Covid's recommendations on TV and in the news. You have refrained from leaving your home, but occasionally go shopping, order deliveries, and have attended some social gatherings. You have started to have symptoms of a cold, such as a stuffy nose and a fever. Since you have heard on television that this may be some symptoms of Covid, you have become very concerned. So you want to know whether it is advisable for you to see a doctor, and in case you are advised to stay at home, how to take care of your health.<br><br>You have recently read a news article about a chatbot developed at UFMG that is intended to provide information on Covid. So you have found it and you are going to interact with it

# Scenario	Patient characteristics	Symptoms	Clinical conditions	Screening	Description
					to see if it helps you get the information you want.<br><br>After the screening process, you will use the app to query for information and ask questions about Covid on topics 4, 9 and any other topic(s) of your choice.
