# Supplementary material for: Evaluating User Experience With a Chatbot Designed as a Public Health Response to the COVID-19 Pandemic in Brazil: Mixed Methods Study
Source: JMIR Hum Factors. 2023 Apr 3;10:e43135. doi: 10.2196/43135 (PMC10131797; doi:10.2196/43135)
Supplement: Multimedia Appendix 2 [file humanfactors_v10i1e43135_app2.pdf]

**Multimedia Appendix 2 - Script for the Interview conducted with asymptomatic (healthy) participants following their interaction with the Chatbot**

The script below is intended to guide a semi-structured interview to be conducted following participants' interaction with the chatbot. It complies with the guidelines in the Underlying Discourse Unveiling Method (UDUM)[1].

| # | Step | Details |
|---|---|---|
| 1) | Consent | ● Read Consent Form, collect and record participant's consent |
| 2) | Participant Data | Collect data on<br>● Age<br>● Education<br>● Occupation<br>● Technology literacy (operating systems and apps regularly used; previous use of chatbot) |
| 3) | Covid experience | How have you been getting information on the disease<br>● Means<br>● Initiative for seeking information<br>Do you live or regularly share living quarters with someone (family members, etc.)<br>Would you mind disclosing whether you belong to a risk group due to any of the following comorbid conditions<br>● Heart disease (angina, heart attack, heart failure)<br>● Lung disease (asthma, bronchitis, emphysema, cystic fibrosis)<br>● Kidney disease<br>● Dialysis<br>● Transplant<br>● Undergoing cancer treatment<br>● Corticosteroid (such as prednisone) or immunosuppressant use (such as cyclophosphamide, azathioprine, methotrexate)<br>● Lupus, rheumatoid arthritis, or other rheumatic disease<br>● Down's syndrome<br>● HIV<br>● Diabetes<br>● Pregnancy or within 4 months after childbirth<br>● Children under 1 year of age<br>● Being elderly<br>Have you or someone close to you ever been infected or suspected of being infected by Covid<br>● Patient Characteristics? |

[1] Semi-structured interview method developed in the field of psychology, but which has been used successfully for the development and evaluation of interactive systems. Reference: Nicolaci-da-Costa, Ana Maria, Carla Faria Leitão, and Daniela Romão-Dias. "Como conhecer usuários através do Método de Explicitação do Discurso Subjacente (MEDS)." VI Simpósio Brasileiro sobre Fatores Humanos em Sistemas Computacionais, IHC (2004): 47-56.

| | | |
|---|---|---|
| | | ● Symptoms?<br>● Medical Conditions? |
| 4) | Information on chatbot | The app to be evaluated has two main functionalities:<br>(i) Providing guidance to people who think they may have Covid, which we refer to as screening;<br>ii) Providing updated information about the disease based on scientific evidence which can be queried through questions. This is grouped into 12 topics ranging from general information, treatment, household guidelines, etc:<br>1. general information<br>2. virus transmission<br>3. disease symptoms<br>4. Guidance for patients with suspected coronavirus infection<br>5. Diagnosis<br>6. Treatment<br>7. Home Care<br>8. Hand and surface hygiene<br>9. Behavior and life habits<br>10. Wearing a mask<br>11. Pregnancy and coronavirus<br>12. Pets |
| 5) | Providing directions | *The purpose of this evaluation is to observe you as you interact with the chatbot, so ideally you should share your screen and, if possible, keep your camera on as well. This part should last about 5 to 10 minutes.*<br><br>*During this first interaction, you will:*<br>*1) pretend that you are a person in an imaginary scenario <scenario allotted to each participant (ex: a pregnant woman who has been having a fever for 1 day>;*<br>*2) Use the chatbot to be screened upon selecting option "I think I'm sick" until you get information on your risk level and guidance;*<br><br>*After that, you will use the chatbot to read updated information in Q&A with the option "read updated information" on at least 3 topics.*<br><br>*Afterwards, you will be asked to answer a few questions about your experience with the chatbot in an interview lasting about 10 to 20 minutes. The session will be recorded to be later transcribed and evaluated by the researchers.*<br><br>*Shall we start?* |

| | | |
|---|---|---|
| 6) | Interaction - Part 1 | Direct observation of participant using the app while being screened through questions |
| 7) | Chatbot experience- Part 1 | **Upon screening:**<br>● What did you think of the process and the final guidance that you were provided?<br>● Would you like to comment on anything in particular? |
| 8) | Interaction - Part 2 | Direct observation of participant using the app while querying for information in the Q&A. |
| 9) | Chatbot experience- Part 2 | **Upon completion of interaction:**<br>● What was your overall impression of your conversation with the chatbot?<br>○ Was it natural?<br>○ Did you feel that at times the chat did not make any sense? If so, when/any example?<br>○ Were there any questions that were not understood by the chatbot?<br>● Positive aspects<br>● Negative aspects<br>● In an actual situation if you had any symptoms or wanted to have more information, would you use this chatbot? Account for your answer.<br>● Any other comments or suggestions?<br><br>The evaluator may also ask questions about any particular moment during the interaction which was observed to be insightful inorder to gain a better understanding of the participant's decisions or perceptions.<br>. |
| 10) | *Acknowledging participant contribution and Closing Statement* | *You have successfully taken part in this evaluation and we thank you very much for your time and contribution.*<br>*Would you like to place any additional comments or questions about this app or the evaluation you have just taken part in?*<br><br><...><br><br>*Thank you very much!* |
