# Supplementary material for: Evaluating User Experience With a Chatbot Designed as a Public Health Response to the COVID-19 Pandemic in Brazil: Mixed Methods Study
Source: JMIR Hum Factors. 2023 Apr 3;10:e43135. doi: 10.2196/43135 (PMC10131797; doi:10.2196/43135)
Supplement: Multimedia Appendix 3 [file humanfactors_v10i1e43135_app3.pdf]

Multimedia Appendix 3 – Table 7 – Complete Version

In the interest of space, Table 7 presented in the paper contained only one example. Here the complete version is presented.

Table 7. Codes, number of occurrences, and examples of negative feedback regarding usability (we translated excerpts into English).

Code	Description	Occurrences, n	Examples
C7. Difficulty in sharing location	Participant unable to share device GPS location when requested by chatbot	10	(Participant starts interacting with the chatbot, until he finds a problem when clicking on the “send location” button the chatbot displays)<br>• P12: What should I do here about this location?<br>• Interviewer: The bot must be requesting whether you allow sharing your location up there, ... When that happens, it sometimes asks if you allow it to share your location, since this is the first time you are using the application.<br>• P12: Oh, ok, ok! It’s because the screen was showing me something else, but now I got it.
C8. Technical problem in mobile app or phone	Chatbot stops responding or gets slow; interaction is interrupted.	4	(P10 started using the chatbot on her mobile phone, when the bot stopped responding for the third time during interaction)<br>• Interviewer: “Right, it should have answered you already, I really don’t know what’s happening...”

			• P10: “Yeah, well, let’s do it on the computer, otherwise we won’t be able to finish this.”<br>• (Participant was able to conclude the scenario on desktop computer)
C9. Need for an option to go back and make a different choice during interaction	Participant needs to start over and repeat entire session, as the chatbot does not have an option for backtracking or choosing a different path during conversation.	2	(P04 inattentively selected the Q&A functionality and had to start over to select the screening one)<br>• Interviewer: You’ve selected the question and answer module, which is a different functionality.<br>• P04: Oh…<br>• Interviewer: We can start with Q&A, or you can start over so you can select screening.<br>• P04: I’d rather start over. Let’s go.
C10. User choice repeatedly prompted by option menu and at high pace	Participant complaint about being prompted to make a choice in option menu and finding it too fast to be able to read the whole answer provided by the bot	6	• “What I didn’t like, but I don’t know if it can be improved, is that the menu prompts you to select an option all the time. This is my feeling about the way the bot operates and not a negative feature of the chatbot.” [P14]

C11. Conversation flow management	Participant does not succeed in keeping the conversation flowing with the chatbot owing to unperceived feedback or lack of it from the chatbot (eg, turn taking management).	4	(Participant selects screening; the chatbot takes a while to respond, indicating that it is typing. In the meantime, the participant sends a new message, typing “yes”)<br>• Interviewer: It seems to me the bot was still typing.<br>• P15: Ok. I’ll wait then ... (Participant comment during interview)<br>• P15: It was difficult until I understood that I had to wait for the bot to answer to keep the conversation flowing.
C12. Better interface resources	Additional features in chatbot interface to enhance interaction.	12	• “Maybe answers should be formatted differently, because then you would clearly distinguish question and answer.” [P10]<br>• “I would suggest clicking on the number, okay? Because I was a little bit confused, [though] you quickly realize what you have to do. But, maybe for convenience, clicking on the number instead of typing it would be easier for me.” [P15]
C13. Insufficient directions on how to interact with the chatbot	Participant requesting directions or help from the interviewer	22	• P01: So, I just have to type inside this little box at the bottom of the screen?<br>• Interviewer: Correct.

			• P15: To be honest, at first I found it difficult to understand what I had to do: I tried to click on the number of the option I wanted to select. Then I realized that I needed to type the number. So that was my first problem using the bot.
C14. Chatbot language need to be adapted to meet different user profiles	Language used by the chatbot needs to be adapted to be understood by user with low literacy level.	2	• "When the app starts you could ask the user's level of education, and if a user reports a low level, the bot may switch to answers that are more adapted to the user's literacy level." [P01]
C15. Chatbot fails to understand unexpected user responses	Chatbot does not successfully process information entered by the participant.	11	• Participant 10 enters "50 years old" in the age field, and the chatbot asks her to enter only a numerical value. The participant then types "50," which is successfully processed by the chatbot and interaction is resumed.
C16. Participants expectations exceed chatbot's actual communicative ability	Participant tries to interact in a way not supported by the chatbot, for example, by trying to speak to the chatbot by voice.	5	• P06: So I should type that I would like to know more about pregnancy? [Participant starts typing "I want to know more about pregnancy"<br>• Interviewer: Actually, each number is a shortcut, you don't have to type everything.

			(The participant tries to interact with the application by voice, and the interviewer notices and guides him)<br>• P15: [starts speaking to his mobile phone] Good afternoon, I would like to have some clarification? Interviewer: our chatbot is not that smart yet, so you will have to type here. You cannot talk to the bot, ok?<br>• P15: Oh, ok.
