# Supplementary material for: Evaluating User Experience With a Chatbot Designed as a Public Health Response to the COVID-19 Pandemic in Brazil: Mixed Methods Study
Source: JMIR Hum Factors. 2023 Apr 3;10:e43135. doi: 10.2196/43135 (PMC10131797; doi:10.2196/43135)
Supplement: Multimedia Appendix 4 [file humanfactors_v10i1e43135_app4.pdf]

Multimedia Appendix 4 – Table 8 – Complete Version

In the interest of space, Table 8 presented in the paper contained only one example. Here the complete version is presented.

Table 8. Codes, number of occurrences (#), and examples of positive feedback regarding health support (we translated excerpts into English).

Code	Description	Occurrences, n	Examples
C17. Outdated information or answer	Participants noticed some outdated information or questioned whether the information presented in the Q&A session was updated.	4	• "How often is the FAQ updated? For instance, whether there's a vaccine or not. ... Because that ensures credibility, right? ... Because sometimes people notice that the information is a little outdated." [P13]
C18. Missing information or explanation	Participants suggested a topic in the Q&A session that should be included or further explained.	5	• "I think there could be some explanation on IgG tests [after reading about IgG tests on one of chatbot's answers]. Because many people have been talking about it and they don't know what that is." [P11]<br>• "I don't know if I got it wrong, although I've read it here, you know, "people with symptoms,

			people...” [Participant starts quoting one of the chatbot’s answers about masks]. But I ... I was in doubt regarding the... I mean, it seemed to me that surgical masks were mandatory only for people who work in the healthcare field, and cloth masks were ok for the rest of the population.” [P13]
C19. Unfulfilled expectations during the screening session	Participants mentioned interesting insights and broken expectations during the screening process	13	(Participant begins the screening session, which ends quickly as she answers “yes” to the first question about shortness of breath [dyspnea])<br>• P12: Is that it?<br>• Interviewer: How did you like the direction you were given?<br>• P12: Well, I said I was short of breath, because normally when I get the flu, I feel short of breath, regardless of COVID. And then, I thought that the direction I got doesn't take this into consideration, right? Because it could really just be the flu and I could be short of breath because of the flu. ... I

			thought it was helpful, because shortness of breath is a worrying symptom, so even if it was a little, it could get worse. But I think there were also other things that should be taken into consideration.<br><br>• P02: ... maybe the person wanted a little bit more information before the chatbot said: "Go to the hospital" [laughs], you know? "Go to the hospital because this is a serious symptom". Maybe something in the sense of reassuring the person, like ... "look, these are symptoms that can be considered ...," ... the direction could be modalized, so as not to scare the person.
C20. Need or demand for actionable orientation during the screening session	Participants expected to receive more practical instructions at the end of the screening session.	5	• “I think there should be something more direct to guide the next step. What am I supposed to do? The bot gave me some explanations about Covid, about my condition, but it didn’t tell me where to go. Given that the person in my scenario is in a risk

			group, as she's pregnant, I thought people would need to know about this." [P06]<br><br>• "Now, if I felt it was a kind of emergency case, the direction was to go to a UPA [local public health center] or to make an appointment with a doctor. Interviewer: Did you find that direction useful? Did it make sense to you?" [P04]<br>• "I may be wrong, but as a person that has diabetes or lives with a person that has diabetes, my first reaction is: what should I do now in an emergency situation? I know I have to go to the hospital and all that stuff, but what can I do here, now, before I get there." [P04]
C21. Demand for situation-oriented answers to questions	Participants expected to find answers that could be more directly applied to a particular situation in the question and answer session.	7	• "Let me comment: my question at the time was how soon I could take the test and what kind of test I should take for a particular situation. And that was my reason for contacting the doctor at the time. I don't know, I think it is not so much the

			goal of the application, but it is also an interesting piece of information. Sometimes people have doubts about which test to take, if they should take the PCR, if they should take the rapid test, depending on how many days they have already had symptoms.” [P07]<br><br>• “If you have traveled, is there a test that allows you to go to your relative's house without having to worry? Or if you actually have to isolate yourself and wait 3 days to see if you won't have anything after leaving the airport?” [P09]
